# Editorial: Advances in molecular biology, pathogenesis, diagnosis, vaccines, and treatment of diseases caused by apicomplexan parasites

**DOI:** 10.3389/fcimb.2023.1205563

**Published:** 2023-04-28

**Authors:** Mingming Liu, Ningbo Xia, William Harold Witola, Kun Li

**Affiliations:** ^1^School of Basic Medicine, Hubei University of Arts and Science, Xiangyang, China; ^2^Guangdong Laboratory for Lingnan Modern Agriculture, College of Veterinary Medicine, South China Agricultural University, Guangzhou, China; ^3^Department of Pathobiology, College of Veterinary Medicine, University of Illinois Urbana-Champaign, Urbana, IL, United States; ^4^Institute of Traditional Chinese Veterinary Medicine, College of Veterinary Medicine, Nanjing Agricultural University, Nanjing, China; ^5^MOE Joint International Research Laboratory of Animal Health and Food Safety, College of Veterinary Medicine, Nanjing Agricultural University, Nanjing, China

**Keywords:** apicomplexan, molecular biology, pathogenesis, diagnosis, vaccines, treatment

Considering the extensive range of diseases caused by apicomplexan parasites, including babesiosis, cryptosporidiosis, leishmaniasis, malaria, neosporosis, and toxoplasmosis, which significantly contribute to the prevalence of fatal parasitic infections, we anticipate that this research area will provide essential insight to aid in the development of innovative strategies for managing and controlling diseases associated with apicomplexan parasites. Finally, thirteen articles were accepted for publication in our Research Topic “*Advances in Molecular Biology, Pathogenesis, Diagnosis, Vaccines, and Treatment of Diseases Caused by Apicomplexan Parasites.*”

Five articles about recent advances in *Toxoplasma gondii* and *Neospora caninum* research have contributed to this Research Topic. Zhang et al. explored the efficacy of panobinostat (LBH589), a novel histone deacetylase inhibitor, against *T. gondii* for treating ocular toxoplasmosis. *In vitro*, LBH589 inhibits proliferation and activity of *T. gondii* in a dose-dependent manner, with low toxicity in retinal pigment epithelial cells. *In vivo*, LBH589 significantly reduced inflammatory cell infiltration and retinal damage in mice while decreasing mRNA expression levels of inflammatory cytokines. These findings suggest that LBH589 holds promise as a preclinical candidate for controlling and curing ocular toxoplasmosis. Yue et al. determined the seroprevalence of *T. gondii* in captive giant pandas and analyzed associated risk factors. Of 203 serum samples collected from 157 pandas between 2007 and 2022, 35.67% were seropositive for *T. gondii*. Age and transfer history between institutions were identified as risk factors, with age-related seroprevalence being the main factor. Housing multiple species together may increase cross-infection risk. These findings provide valuable data for creating policies to prevent and control *T. gondii* infections, protecting the health of captive giant pandas and other wildlife. Qi et al. developed serological testing methods using TgSAG1, TgGRA7, and TgBAG1 proteins to detect *T. gondii*-specific immunoglobulin M and immunoglobulin G antibodies in 3,733 animals from the Qinghai Tibetan Plateau. These methods, including rSAG1-ELISA, rGRA7-ELISA, and rBAG1-ELISA, also differentiated between acute and chronic toxoplasmosis infections. The study confirmed that SAG1, GRA7, and BAG1 recombinant antigens can effectively be used to detect specific antibodies and distinguish between acute and chronic *T. gondii* infections, providing significant clinical evidence for toxoplasmosis diagnosis. Zou et al. used high-throughput RNA sequencing to study expression of circRNAs and miRNAs in the liver of mice infected with *T. gondii* during acute and chronic stages. They found 265 and 97 differentially expressed (DE) circRNAs and 171 and 77 DEmiRNAs at acute and chronic stages, respectively. One DEcircRNA showed a significant correlation with two DEmiRNAs in a network associated with liver immunity and disease pathogenesis. These findings help in understanding how circRNA expression in the liver changes after *T. gondii* infection and improve knowledge of hepatic toxoplasmosis in mice. Fereig et al. investigated the prevalence of *T. gondii* and *N. caninum* in camels imported from Sudan for human consumption and found seropositive rates of 25.7% for *T. gondii*, 3.9% for *N. caninum*, and 0.8% for mixed infections in 460 camels. The study also revealed variations in infection rates by region. A systematic review showed an overall global seroprevalence of 28.6% for *T. gondii* and 14.3% for *N. caninum* in camels. These findings provide important information to guide control and prevention strategies for these parasites in camels.

Four articles have been published on this Research Topic, focusing on the current progress in *Babesia* and *Plasmodium*. BmGPI12 which serves as a reliable biomarker for active *B. microti* infection. Chand et al. characterized 18 monoclonal antibodies against BmGPI12, identifying five unique epitopes through serological profiling and competition assays. They found five antibody combinations that specifically detected the secreted form of BmGPI12 in plasma samples from infected mice and humans. This finding may contribute to the development of improved diagnostic tools for human babesiosis. Ji et al. investigated the effectiveness of continuous PI4K-targeting treatment for babesiosis in immunocompromised individuals. *B. microti*-infected SCID mice were treated with MMV390048, a PI4K inhibitor, for 72 days. PCR tests were negative from 64 days. The study also found an atovaquone-resistant *B. microti* strain with a Y272C mutation in the Cytb gene. Significantly, MMV390048 effectively inhibited this resistant strain. The findings suggest that PI4K inhibitors may be a promising therapeutic option for treating human babesiosis. Zhou et al. investigated the mechanisms behind thrombocytopenia and anemia in malaria and babesiosis, demonstrating that infection by *Babesia* and *Plasmodium* species stimulate production of anti-erythrocyte and anti-platelet autoantibodies, with B and T lymphocytes playing a significant role in their production. Membrane-associated cytoskeleton proteins might also influence generation of these autoimmune antibodies. The study suggests that an autoimmune response mediated by autoantibodies contributes to thrombocytopenia and hemolytic anemia and plays a role in regulating the overall autoimmune response in these infections. The malaria vaccine candidate BK-SE36 is based on *P. falciparum* SERA5. Despite promising results in clinical trials, concerns about genetic diversity and allele-specific immunity persist. Arisue et al. found polymorphisms in sera5 to be primarily in repeat regions and identified a consensus sequence with African-specific variations. There was no significant genetic differentiation between parasites in vaccinated and control groups, suggesting that the vaccine does not trigger an allele-specific immune response.

Three articles on *Cryptosporidium* research have also contributed to this Research Topic. Khan and Witola discuss the urgent need for effective anti-*Cryptosporidium* drugs, as the current FDA-approved drug nitazoxanide has limited efficacy in immunodeficient patients, young children, and neonatal livestock. The authors provide an overview of past and present pharmacotherapy in humans and animals for *Cryptosporidium* infections, highlighting progress in the field and discussing various strategies employed for discovering and developing effective treatments for cryptosporidiosis. Dong et al. conducted a study on *Cryptosporidium*-infected and healthy yaks, analyzing 16S rRNA sequencing and short-chain fatty acid (SCFA) concentrations. The results revealed significant differences in the abundance of phyla, genera, enzymes, and pathways between the two groups. Infected yaks also showed notably lower concentrations of various SCFAs. The study suggests that *Cryptosporidium* infection leads to gut dysbiosis and a decrease in SCFA concentrations, offering potential insight for preventing and treating diarrhea in livestock. Li et al. conducted a meta-analysis of 35 articles published before 2021 to determine the global prevalence of *Cryptosporidium* in *Equus* animals, finding an overall prevalence rate of 7.59%. Higher rates were observed in younger and female animals, with the highest prevalence in scale breeding *Equus*. *C. muris* was the most common genotype detected, and low altitude, rainy, humid, and tropical climates were associated with higher prevalence rates. To reduce infection, farmers should focus on managing young and female *Equus* animals, improving water filtration systems, reducing stocking densities, and treating livestock manure.

A study on *Leishmania* was contributed by Khanra et al. They examined three clinical isolates to identify common features in genetically diverse antimony (Sb)-resistant *Leishmania* parasites. The research found that resistant isolates had significantly higher intracellular thiol content and expression of thiol-synthesis genes compared to sensitive isolates. Additionally, resistant isolates had increased expression of Sb-reducing enzymes and Sb transporter genes. This study suggests that diverse Sb-resistant parasites develop a resistant phenotype by enhancing thiol synthesis and Sb transporter gene expression.

In summary, this Research Topic has provided valuable insight into recent advancements in the study of apicomplexan parasites, each of which warrants further investigation to improve both human and animal health outcomes.

## Author contributions

ML organized and wrote the editorial. NX, WW and KL revised it. All authors contributed to the article and approved the submitted version.

